# Unraveling the Systemic and Local Immune Response of Rainbow Trout (*Oncorhynchus mykiss*) to the Viral Hemorrhagic Septicemic Virus

**DOI:** 10.3390/biology14081003

**Published:** 2025-08-05

**Authors:** Mariana Vaz, Gonçalo Espregueira Themudo, Felipe Bolgenhagen Schöninger, Inês Carvalho, Carolina Tafalla, Patricia Díaz-Rosales, Lourenço Ramos-Pinto, Benjamín Costas, Marina Machado

**Affiliations:** 1 Centro Interdisciplinar de Investigaçao Marinha e Ambiental (CIIMAR), University of Porto, Terminal de Cruzeiros do Porto de Leixões, Av. General Norton de Matos s/n, 4450-208 Porto, Portugal; gthemudo@ciimar.up.pt (G.E.T.); fschoninger@ciimar.up.pt (F.B.S.); maria.carvalho@ciimar.up.pt (I.C.); lourenco.pinto@ciimar.up.pt (L.R.-P.); bcostas@ciimar.up.pt (B.C.); 2 Faculty of Mathematics and Natural Sciences, University of Bergen, 5007 Bergen, Norway; 3 Fish Immunology and Pathology Group, Biotechnology Department, National Institute for Agricultural and Food Research and Technology (INIA), Spanish National Research Council (CSIC), Carretera de La Coruña km 7.5, 28040 Madrid, Spain; tafalla@inia.csic.es (C.T.); pdiazrosales@inia.csic.es (P.D.-R.); 4 Instituto de Ciências Biomédicas Abel Salazar (ICBAS-UP), University of Porto, Rua de Jorge Viterbo Ferreira 228, 4050-313 Porto, Portugal

**Keywords:** immune response, viral load, antiviral, viperin, transcriptomics

## Abstract

Viral outbreaks have caused significant mortality and economic losses in aquaculture, highlighting the urgent need for effective therapies and a deeper understanding of antiviral and immune mechanisms in key species. This study investigates the constitutive and virus-induced antiviral responses in juvenile rainbow trout (*Oncorhynchus mykiss*) following infection with viral hemorrhagic septicemia virus (VHSV). Trout (30 g) were infected by immersion with VHSV (TCID_50_ = 10^5^ mL^−1^) for two hours. Samples were collected at 24, 72, and 120 h post-infection to assess hematology, innate immunity, viral load, and transcriptomic response. At 24 h post-infection, no immune response or increase in viral load was detected, suggesting the host had not yet recognized the virus and was still in the incubation phase. By 72 h, viral replication peaked, with high viral loads observed in mucosal tissues (skin and gills) and immune organs (kidney, spleen, liver), alongside strong up-regulation of antiviral genes, such as *viperin*. This gene maintained high expression through the final sampling point, indicating its key role in the antiviral response. At this stage, reduced immune competence was observed, marked by elevated nitric oxide and circulating thrombocytes. At 120 h, modest increases in peripheral monocyte, plasma lysozyme, and peroxidase activity were detected; however, these responses were insufficient to reduce viral load, suggesting the resolution phase had not yet begun. In summary, while a limited immune response was observed by the end of the trial, the consistent antiviral activity of *viperin* from peak infection to 120 h post-infection underscores its importance in the defence against VHSV in rainbow trout.

## Simple Summary

Viral diseases are a major problem in fish farming, causing high death rates and serious economic losses. One such disease, caused by a virus known as VHSV, affects rainbow trout, a widely farmed fish. In this study, we explored how young rainbow trout respond to this virus over time. The fish were infected in a controlled environment, and their immune responses were observed at different stages. In the early phase, the fish showed no signs of fighting the virus, likely because the virus had not yet been detected by their immune system. After 72 h, the virus had spread to several organs, including the skin, gills, and immune-related tissues, and signs of a strong immune response began to appear. A specific gene called viperin, which helps combat viruses, was highly active during this stage. However, 120 h post-infection, although there were some signs that the immune system was reacting, the virus was still present in high amounts. These findings help us better understand how rainbow trout respond to viral infections and highlight the importance of certain immune mechanisms. This knowledge can help develop more effective treatments and strategies to protect production against infectious outbreaks, improving welfare in aquaculture.

## 1. Introduction

### 1.1. Impact of VHSV Infections

The intensification of production systems in aquaculture has significantly contributed to the high incidence of pathological infections caused by bacteria, viruses, and parasites. Viral outbreaks, in particular, have a substantial economic impact on the aquaculture industry, highlighting the urgent need for the development of both prophylactic and therapeutic solutions to support effective animal health management. According to the 2014 Aquatic Animal Health Code of the OIE (World Organization for Animal Health), viruses are responsible for 8 of 10 diseases with significant social, economic, and public health repercussions, and they pose a potential risk to the aquaculture sector. This surge in infections has resulted in high mortality rates, leading to considerable economic losses [1], and justifies the pressing need for more efficient therapeutic strategies, aligned with the critical need to deepen our understanding of antiviral responses and the immune mechanisms of target species. Viral hemorrhagic septicemia virus (VHSV), an enveloped negative-sense single-stranded RNA (ssRNA) virus composed of six genes encoding phosphoprotein (P), nucleoprotein (N), glycoprotein (G), non-virion protein (NV), matrix protein (M), and RNA-dependent RNA polymerase (L), belongs to the *Novirhabdovirus* genus, within the Rhabdoviridae family [2,3,4]. This virus replicates through RNA-dependent RNA synthesis, in which a unique class of RNA polymerases known as RNA-dependent RNA polymerases (RdRps) are employed [5,6].

### 1.2. Symptomatology

VHSV is associated with high mortality rates—reaching up to 90%—at water temperatures below 15 °C [4,7,8,9], affecting a wide range of freshwater and marine fish species in aquaculture, particularly juvenile rainbow trout (*Oncorhynchus mykiss*) [10], turbot (*Scophthalmus maximus*) [11], and olive flounder (*Paralichthys olivaceus*) [4,12]. VHSV targets the endothelial cells of blood vessels, leading to severe hemorrhaging in the skin and internal organs [4,13]. Before actively reaching the blood vessels, this virus uses the gills and skin as external tissues to enter the fish. These organs allow for the uptake and replication of VHSV [14,15] and then dissemination to other tissues through the bloodstream [15]. Infected fish may present numerous non-specific clinical signs in the early stages of infection, such as darkening of the skin; hemorrhage in the fins, gills, mouth, eyes, and skin; exophthalmia; anemic gills; lethargy; ascites; and distention of the abdomen [16]. In addition to these symptoms, this virus is able to reach the central nervous system, inducing abnormal swimming behavior [16]. The liver and hematopoietic tissues (spleen and head kidney) are also affected [17]. The concomitant damage caused by the infection can lead to high mortality rates and, consequently, to significant economic losses in aquaculture.

### 1.3. Host Antiviral Response

When infection occurs, virus clearance is dependent on the host cell antiviral protein synthesis machinery. The efficacy of the viral replication within the host cells is related to the pathogen’s ability to evade the fish immune response by inhibiting the expression of interferons (IFNs) while the host cell synthesizes their viral products [18]. It is known that the VHSV antigen detection triggers the rapid induction and activation of IFN transcription, as it is highly sensitive to host cell type I IFNs responses during the early stages of replication [4,18]. The these secreted antiviral cytokines [19] bind to specific cell surface receptors, triggering the expression of numerous interferon-stimulated genes (ISGs), including Mx (myxovirus resistance), viperin (VIP, virus inhibitory protein, endoplasmic reticulum-associated, interferon-inducible, also known as RSAD2 (radical S-adenosyl methionine domain-containing protein 2)), IRFs (interferon regulatory factors), and ISG15 (interferon-stimulated gene 15). These antiviral proteins directly respond to VHSV infection [20], playing a crucial role in limiting viral replication by inducing an antiviral state within the cell and activating the innate immune response [21,22,23,24].

### 1.4. Viperin Mode of Action

Viperin, a SAM (S-adenosyl-L-methionine) radical-dependent enzyme, is characterized as having an important role in innate immune signaling and response [25,26]. The catalytic activity of viperin was recently elucidated through its ability to synthesize the antiviral ribonucleotide 3′-deoxy-3′,4′-didehydrocytidine triphosphate (ddhCTP), which inhibits the replication of various RNA viruses. This inhibition occurs via chain termination when ddhCTP is incorrectly incorporated by viral RNA-dependent RNA polymerases [27]. The synthesis of the antiviral ribonucleotide ddhCTP provides a mechanistic explanation for how viperin suppresses the replication of certain viruses, notably VHSV, and also sheds light on how this antiviral protein interacts with other cellular proteins. These interactions are now understood to be essential for the broad-spectrum antiviral activity associated with viperin expression [28]. It is increasingly evident that, in animals, viperin is integrated into the wider cellular antiviral response through a complex network of protein–protein interactions. Among the various antiviral properties attributed to viperin, it has been shown to promote protein degradation at both the cellular and viral levels [28].

In fact, in recent years, studies have fully elucidated the biological function of VIP, highlighting its pivotal role as an antiviral defense in mammals and Atlantic cod (*Gadus morhua*) and grass carp (*Ctenopharyngodon idella*) [24,29]. While the first VIP homolog was discovered in rainbow trout [30,31], no further studies have been carried out on the matter in this species. The limited understanding of host–pathogen interactions and the systemic and local immune responses of trout to VHSV underscores the need for further investigation. Therefore, the present study aims to comprehensively evaluate both constitutive and induced antiviral mechanisms in juvenile rainbow trout in response to VHSV infection.

## 2. Materials and Methods

### 2.1. Experimental Design and Infection Method

The study was conducted at Interdisciplinary Centre of Marine and Environmental Research (CIIMAR, University of Porto) in accordance with “The guidelines on animals protection used for scientific purposes from the European Directive 2010/63/EU”, reviewed and approved by 0421/000/000/2020 and all procedures were performed by trained scientists (following FELASA category C recommendations).

Forty-two rainbow trout (*Oncorhynchus mykiss*) juveniles were obtained from a local trout farm (Serra D’aires, Portugal) with no history of VHSV outbreaks. Trout with an average initial weight of 30 g were randomly distributed in two independent water recirculation systems composed of three tanks each (50 l tanks, 7 fish per tank) with the following conditions: temperature of 18.53 ± 0.75 °C, photoperiod automatically controlled (14 h light/10 h dark), dissolved oxygen at 8.12 ± 0.18 mg L^−1^, ammonia 1.25 ± 0.01 and nitrite 0.58 ± 0.11 mg L^−1^. The temperature used in this trial allows mortality to be reduced or non-existent, but with high levels of replication [1]. After an acclimatization period of one week, the water recirculation of both systems was stopped, and fish from one system were challenged by immersion (2 h bath with strong aeration), with a concentration of TCID_50_ = 10^5^ mL^−1^ of viral hemorrhagic septicemia virus (VHSV, strain 0771). The control (uninfected) group was exposed to the same bathing procedure but without the virus. Water recirculation from the second system was also stopped to mimic the same bath-associated conditions to serve as a control (non-infected fish). After the 2 h bath period, water recirculation from both systems was restored.

Two fish per tank (n = 6 per treatment/time) were sampled at 24, 72, and 120 h post-infection (infected and uninfected groups). Fish were euthanized by administering an overdose of anesthetic (1 mL, 2-phenoxyethanol; Sigma-Aldrich, Saint Louis, MO, USA). Fresh blood samples were collected from the caudal vein for hematological analysis, while plasma was isolated and stored at −80 °C for immune response measurements. Tissues, including the skin, gills, head-kidney (HK), spleen, liver, and gut, were harvested and immediately frozen in liquid nitrogen and posteriorly stored at −80 °C for viral load. Additionally, gills, HK, and spleen were also preserved in RNAlater (GTRADV-500, GenTegra, Pleasanton, CA, USA) (with a proportion of 1/10 *w*/*v*), kept at 4 °C for the first 24 h and then stored at −80 °C until processing for RNA sequencing.

### 2.2. Hematological Parameters and Differential Peripheral Leukocyte Counts

The hematological profile was carried out from fresh blood collected from the caudal vein with heparinized syringes. The hematological profile consisted of total red blood cell (RBC), white blood cell (WBC), and hemoglobin concentration (Drabkin colorimetric method; SPINREACT, 1001230, Girona, Spain) and hematocrit values. After this, the blood was centrifuged (10,000× *g* for 10 min at 4 °C) for plasma collection. The plasma collected was stored at −80 °C until analysis.

For blood differential leukocyte count, blood smears were prepared from fresh blood, air-dried, and later fixed with an ethanol–formaldehyde solution (90% absolute ethanol with 3.7% formaldehyde) for 1 min [32]. Neutrophil staining was performed in accordance with Afonso et al. [33], and then the slides were stained with hematoxylin and eosin. Two hundred leukocytes were counted, from which percentages of neutrophils, lymphocytes, monocytes, and thrombocytes were counted. Blood indices, mean corpuscular volume (MCV), mean corpuscular hemoglobin (MCH) and mean corpuscular hemoglobin concentration (MCHC) were determined according to Machado et al. [34].

### 2.3. Plasma Innate Immune Response

Antiproteases activity. Plasma antiprotease activity was determined by the percentage of inhibited trypsin by plasma proteases [35]. A volume of 10 µL of plasma was used with 10 µL of trypsin solution (in NaHCO_3_, 5 mg mL ^–1^, Sigma T-0604, 5G), which was incubated at room temperature (RT) for 10 min. After the incubation time, 125 µL of azocasein (20 mg mL^–1^) in NaHCO_3_ and 100 µL of phosphate-buffered solution (PBS, NaH_2_PO_4_, 13.9 mg mL^−1^, pH 7.0) were added, and this mixture was incubated for 1 h at RT. Thereafter, the reaction was stopped with the addition of 250 µL of trichloroacetic acid (TCA) (100 mg mL^−1^, Sigma T-08657, 250G). Samples were read at 450 nm, and readings were determined using a reference control, which includes trypsin, azocasein, and PBS.

Proteases activity. Proteases activity was measured in plasma according to [31]. To each 10 µL of plasma was added 125 µL of azocasein in NaHCO_3_ (20 mg mL^−1^, Sigma A-2765, 5G) and after 100 µL of PBS (NaH_2_PO_4_, 13.9 mg mL^−1^, pH 7.0) was added and incubated for 24 h at room temperature. The reaction was stopped with 250 µL of trichloroacetic acid (TCA) (100 mg mL^−1^, Sigma T-08657, 250G). Samples were read at 450 nm, and readings were determined using a reference control, which includes azocasein, PBS and trypsin.

Lysozyme. A turbidimetric assay was used to evaluate lysozyme according to [36]. A bacterial solution of *Micrococcus lysodeikticus* (0.25 mg mL^−1^, in Na_2_HPO_4_ 0.05 M, with pH 6.2) was prepared. Plasma samples (10 µL in triplicates) were added to 150 µL of bacteria solution in a microplate. A standard curve was prepared with serial dilutions of lyophilized hen egg white lysozyme (Sigma, EUA) in Na_2_HPO_4_ (0.05 M, pH 6.2). The reaction was carried out at RT, and the absorbance (450 nm) was measured after 0 and 20 min. The concentration of lysozyme in the samples was calculated using the formula of the standard curve.

Peroxidase activity. Plasma peroxidase activity was determined according to [37]. Five microliters of plasma were diluted in triplicates with HBSS (Hank’s Balanced Salt Solution) without Ca^2+^ and Mg^2+^ (Cytiva, Marlborough, MA, USA) in a microplate, for a final volume of 150 µL. Then, 50 µL of 3,3′,5,5′-tetramethylbenzidine hydrochloride (TMB 20 mM, Sigma, EUA) and 50 µL of H_2_O_2_ (5 mM) were added. After 2 min, the reaction was stopped with the addition of 50 µL of H_2_SO_4_ (2 M). Wells without plasma were used as blanks. The plasma peroxidase activity was determined at 450 nm by defining one unit of peroxidase as the amount that produces an absorbance change of 1 OD (expressed in units mL^−1^ of plasma).

Nitric oxide. The amount of nitric oxide (NO) in plasma was determined using the nitrite/nitrate colorimetric method kit (Roche Applied Science, 11746081001, Basel, Switzerland). The supplier’s instructions were followed. Plasma samples were diluted 1:10 in distilled water, and a calibration curve was obtained with serial dilutions of sodium nitrite (500 mg L^−1^).

Bactericidal activity. For bactericidal activity, *Photobacterium damselae* subsp. *piscicida* (M1415) was used in tryptic soy agar (TSA) medium supplemented with 1.5% of NaCl (TSA saline, TSAs) overnight at 25 °C. After inoculation, colonies were scraped off with a loop and dissolved in tryptic soy broth (TSB saline, TSBs) supplemented with 1.5% of NaCl and incubated overnight at 25 °C. Then, the optical density at 600 nm (desired: 0.200–0.300) was determined. Twenty microliters of each sample were pipetted into the U-shaped microplate, including the positive control (HBSS with Ca^2+^ and Mg^2+^, without phenol) and negative control (TSBs). Then, 20 µL of bacteria were added to the samples and the positive control and incubated for 2.5 h at 25 °C with shaking. A 25 µL measure of thiazolyl blue tetrazolium bromide (MTT) (1 mg mL^−1^, Sigma, EUA) was added, and the plates were incubated for 10 min at 25 °C with shaking. Then, the plates were centrifuged (2000× *g* for 10 min) and the supernatant was discarded. Two hundred microliters (200 µL) of dimethyl sulfoxide (DMSO, Sigma, EUA) was added to 100 µL of the solution and then transferred to a flat-bottom plate, and the activity was read at 560 nm, following the method described by Graham and Secombes [38], with modifications [34,39].

All analytics were performed in triplicate.

### 2.4. Viral Load

RNA was extracted from the virus, skin, gills, liver, gut, HK and spleen using and following the instructions of the RNA isolation and DNase treatment kit (NZY Total RNA Isolation Kit (NZYTech, MB13402, Lisbon, Portugal). RNA samples from gills, HK and spleen were also used for RNAseq analysis. The RNA samples were quantified by spectrophotometry using DeNovix DS-11 FX (Wilmington, DE, USA), and integrity was verified in a 2% agarose gel electrophoresis. Then, the RNA samples and viral RNA were transformed into cDNA using the QuantiTect^®^ Reverse Transcription Kit (Qiagen, 205311, Düsseldorf, Germany). VHSV quantification was performed with iTaq^TM^ Universal Probes Supermix (Bio-Rad, Hercules, CA, USA) with a final volume of 10 µL. The primers used were VHSV-N-for (5′-GACTCAACGGGACAGGAATGA-3′) and VHSV-N-rev (5′-GGGCAATGCCCAAGTTGTT-3′) and a specific probe for VHSV (TGGGTTGTTCACCCAGGCCGC) 5′-end with the reporter molecule 6-carboxy fluorescein (FAM) and at the 3′-end with the quencher 6-carboxytetramethyl-rhodamine (TAM) [40], at a final concentration of 10 µM. The samples and standard curve were pipetted in triplicate. The standard curve was prepared with 10-fold serial dilutions [36]. The CFX384^TM^ Real-Time System (Bio-Rad, CA, USA) was used with the following conditions: polymerase activation and DNA denaturation 10 sec at 95 °C and 40 cycles of 5 s at 95 °C and 30 s at 60 °C. With the threshold cycle (Ct) obtained, the amount of virus in the samples were calculated by using the equation of the line [x = (yc)/m] determined with the standard curve Ct values plotted against TCID_50_.

### 2.5. Gills, Skin and HK Tissue RNA Extraction and Sequencing

Total RNA was extracted from gills, HK and spleen, as previously mentioned. Random samples were selected for each experimental group (n = 3, with each replicate consisting of a pool of two fish from the same tank), at several sampling times (24, 72 and 120 h after infection) and total RNA extracted with the NZY Total RNA Isolation kit (NZYTech, Lisbon, Portugal) according to the manufacturer’s instructions after tissue homogenization in TRIzol Reagent (Invitrogen, St. Louis, MO, USA). The RNA integrity was verified through gel electrophoresis, and its purity and concentration were quantified using Qubit™ RNA BR (Broad-Range) Assay Kits (Thermo Fisher Scientific, Q10210, Pittsburgh, PA, USA) following the instructions. Total RNA was sent to the company Novogene (Cambridge, UK) for sequencing.

Sequencing was performed using Illumina’s TruSeq stranded mRNA kit (San Diego, CA, USA) and sequenced on an Illumina Novaseq 6000 instrument (San Diego, CA, USA) as PE150 reads, and a total of 12 cDNA libraries were built (3 of each treatment). The quality of sequencing data was evaluated using FastQC v0.11.8 (https://www.bioinformatics.babraham.ac.uk/projects/fastqc/) (accessed on 20 October 2023), and reads with low quality (Phred score < 15 and read length < 30 bp) were removed using Fastp v0.23.4 [41]. The remaining reads were pseudo-aligned to the rainbow trout reference transcriptome (USDA_OmykA_1.1) with Kallisto v0.46.0 [42]. Transcript expression levels were imported into R v4.3.1 via the R/tximport v1.30.0 package. Comparisons were made using the DEseq package (v.1.44.0) against the CTRL data (non-infected), which served as the reference group. Genes with False Discovery Rate (FDR)-adjusted *p*-values ≤ 0.01 and an absolute Log2 Fold Change (Log2FC) ≥ 2 compared to the CTRL group were classified as differentially expressed genes (DEGs).

The results and Gene Ontology (GO) enrichment analysis were conducted using the g:Profiler web tool (version e111_eg58_p18_f463989d, https://biit.cs.ut.ee/gprofiler/gost) (accessed on 11 November 2023). Gene Ontology (GO) terms were analyzed and categorized into biological processes (BPs), cellular components (CCs), and molecular functions (MFs), applying the Benjamini–Hochberg FDR method with a significance threshold of *p*-value < 0.05. The bubble charts displaying the enriched GO terms were generated using RStudio (version R 4.2.1). Gene names associated with the enriched GO terms were obtained through the Ensembl BioMart tool (https://www.ensembl.org/index.html) (accessed on 20 October 2023).

### 2.6. Statistical Analysis

Except for RNA-seq, results are expressed as mean ± standard deviation (mean ± SD). Data were analyzed for normality and homogeneity of variance and, when necessary, transformed before being treated statistically. All data was analyzed by two and three-way ANOVA (for viral load), with time and infection as factors, followed by a Tukey post hoc test to identify differences within time. Statistical significance was tested at *p* < 0.05, and all statistical analyses were performed using STATISTICA 12 software for WINDOWS.

## 3. Results

### 3.1. Hematological Parameters in Response to Infection

The hematological response of rainbow trout infected with VHSV is presented in Table 1. No statistically significant differences (*p* > 0.05) were observed in total WBC, MCH, MCHC and neutrophil counts. The concentrations of RBCs and hemoglobin were found to be reduced (*p* < 0.01) at 24 h in fish exposed to the infection. At 120 h, a significant reduction (*p* = 0.041) in the hematocrit levels was found in infected fish compared to the CTRL group. Regardless of time, infected fish showed a lower concentration of peripheral lymphocytes compared to non-infected ones. Moreover, challenged fish showed a significantly higher concentration of peripheral monocytes at 120 h compared to the first sampling (24 h), while the number of thrombocytes remained high in the first two sampling points with a significant drop at 120 h (*p* = 0.03). Nonetheless, MCV increased (*p* = 0.05) at 120 h compared to 24 h while hemoglobin decreased at 72 and 120 h compared to the 24 h sampling, regardless of infection status.

### 3.2. Innate Immune Response

**Figure 1:**
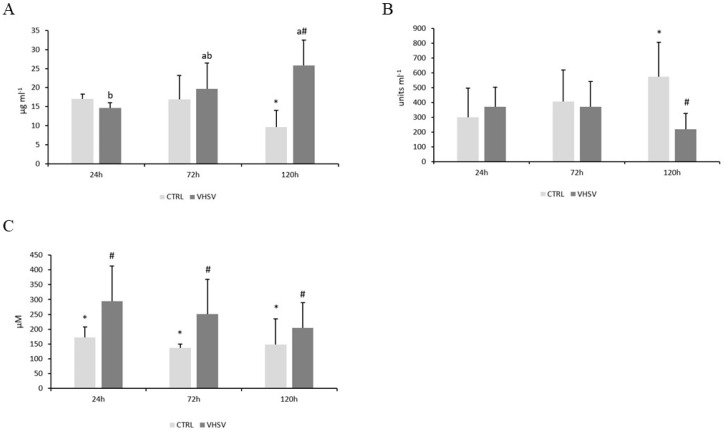
Lysozyme concentration (**A**), peroxidase activity (**B**), and NO concentration (**C**) of rainbow trout challenged with VHSV and sampled 24, 72 and 120 h post-infection. Values represent means ± SD (n = 6). Different letters stand for significant differences attributed to time. Different symbols stand for significant differences attributed to infection (CTRL vs. VHSV). (Multifactorial ANOVA; Tukey post hoc test; *p* ≤ 0.05.) CTRL (control group) and VHSV (infected group).

Figure 1 shows the immune response in plasma of rainbow trout during the infection times. No statistically significant differences (*p* > 0.05) were observed in antiproteases, proteases or bactericidal activities (Supplementary Materials Figure S1A, Figure S1B and Figure S1C, respectively). The plasma lysozyme concentration (Figure 1A) showed a significant increase (*p* < 0.01) at 120 h post-challenge compared to the control, and peroxidase activity (Figure 1B) behaved in the opposite way (*p* = 0.03). When exposed to VHSV, NO production (Figure 1C) was significantly higher, regardless of time.

### 3.3. Viral Load

A standard curve using cDNA VHSV dilutions was prepared to obtain one linear regression between Ct mean values and VHSV concentration used for bath infection (TCID_50_ = 10^5^ mL^−1^) (Supplementary Figure S2). Therefore, quantification of viral load in tissues was determined according to the following equation: Y=−3.1357x+ 45.975, with R^2^ = 0.9909.

**Figure 2:**
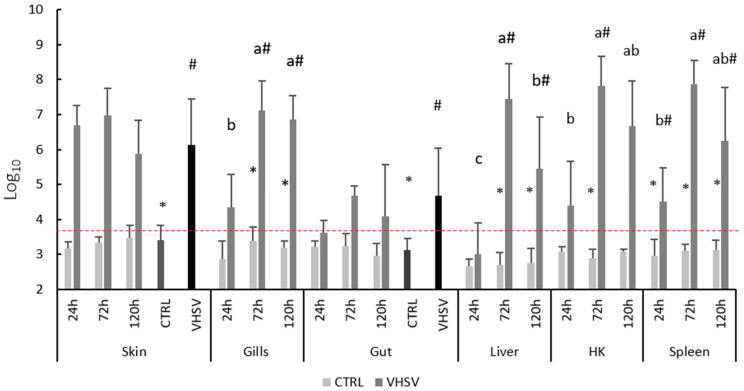
Viral quantification in skin, gills, gut, liver, HK and spleen of rainbow trout challenged with VHSV and sampled at 24, 72 and 120 h post-infection. Different letters stand for significant differences attributed to time. Different symbols stand for significant differences attributed to infection (CTRL vs. VHSV). (Multifactorial ANOVA; Tukey post hoc test; *p ≤* 0.05).

Figure 2 presents the viral load determination in skin, gills, gut, liver, HK and spleen. It is important to highlight that VHSV was not detected in any of the CTRL groups (red line represents the basal level of expression). When infected and regardless of the tissue, the presence of viral particles was observed. In skin (10^6.97^), gills (10^7.12^), liver (10^7.43^), HK (10^7.83^) and spleen (10^7.87^), the viral load peak was detected at 72 h, with significantly higher levels compared to those observed at 24 h in gills, liver, HK and spleen. Skin and gut tissues failed to display the same pattern. Additionally, a significant decrease in the viral copy number at 120 h was observed in the liver (10^5.45^). No mortality was observed.

### 3.4. Tissue RNAseq

#### 3.4.1. Differential Expression Analysis

**Figure 3:**
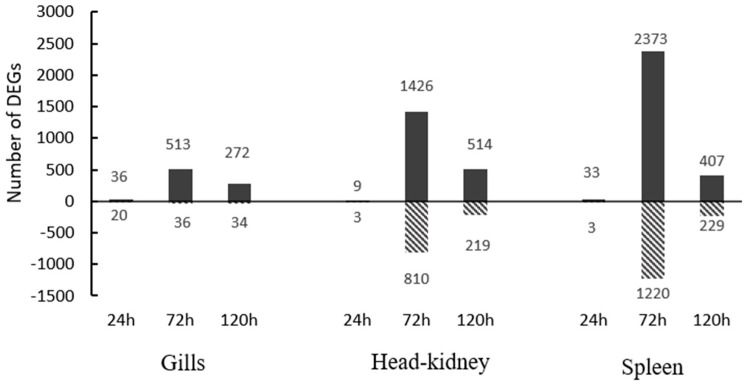
Diverted stacked bar chart showing differentially expressed genes (DEGs) up and down-regulated in gills, HK and spleen in rainbow trout at 24, 72 and 120 h post-infection with VHSV.

Gills, HK and spleen samples collected before infection and at 24, 48 and 72 h post-infection were subjected to RNA sequencing. The differential expression analysis was then analyzed between pre-infected and infected samples at each time. This analysis revealed a peak in the number of both up- and down-regulated differential expressions of genes (DEGs) at 72 h in all analyzed tissues (Figure 3), followed by the 120 h and 24 h. At this time (72 h), the number of up- and down-regulated DEGs in the HK and spleen was significantly higher than in the gills, with the spleen being the tissue with the highest number of up- and down-regulated genes.

#### 3.4.2. Enrichment Analysis

**Figure 4:**
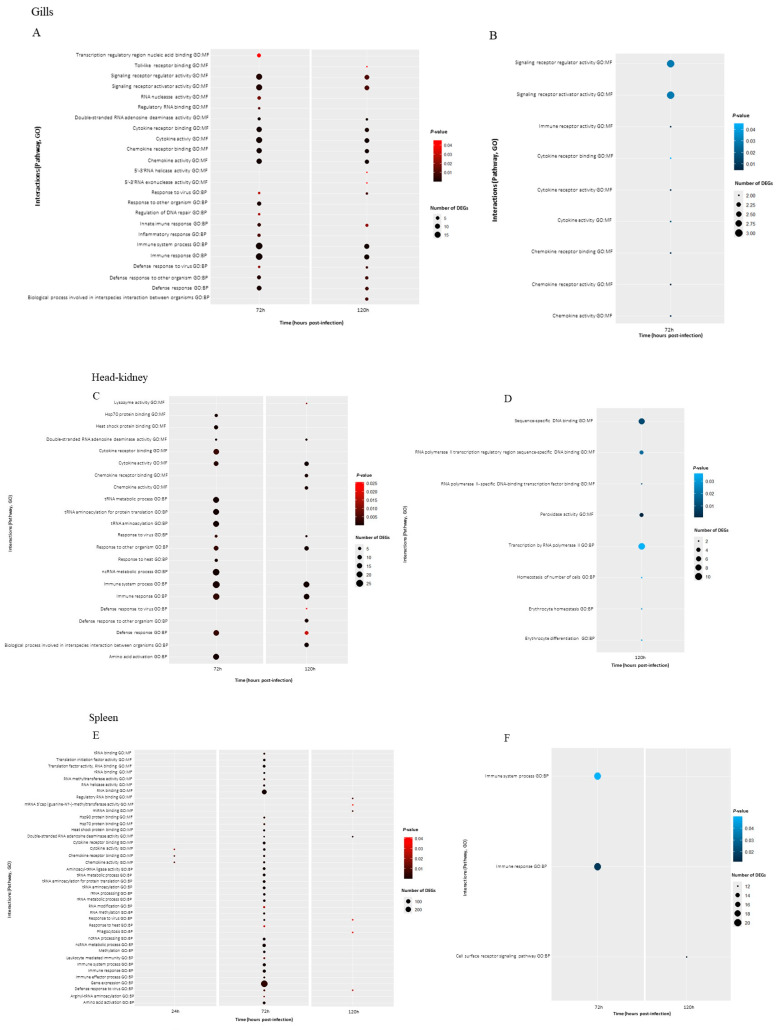
Bubble charts of the gene ontology (GO) enrichment analysis in gills, HK and spleen of rainbow trout at 24, 72 and 120 h post-infection with VHSV. (**A**) Gills up-regulated; (**B**) gills down-regulated; (**C**) HK up-regulated; (**D**) HK down-regulated; (**E**) spleen up-regulated and (**F**) spleen down-regulated. MF: molecular function; BP: biological process.

To further investigate the functional implications of DEGs, Gene Ontology (GO) enrichment analyses were performed on the significantly up-regulated and down-regulated DEGs for each tissue. Figure 4 presents bubble plots of the GO enrichment analysis of immune-related processes in the gills, HK, and spleen of rainbow trout at 24, 72 and 120 h post-infection with VHSV. Furthermore, due to the reduced number of DEGs at 24 h in the gills, spleen and HK, no enriched GO terms were obtained. The lack of enriched and down-regulated GO terms in the gills at 120 h and in the HK at 72 h also did not allow the identification of genes commonly expressed at these times and in comparison with the other time points.

In the gills (Figure 4A, Supplementary File S1) several molecular functions (MFs) and biological processes (BPs) were identified in common and positively enriched at 72 h and 120 h after infection, such as MF: “signalling receptor regulator/activator activity”, “cytokine receptor binding”, “cytokine activity”, “chemokine receptor binding”, “chemokine activity”, BP: “response to virus”, “innate immune response” “innate system process”, “innate response”, “defense to virus”, “defense response to other organism” and “defense response”. Exclusively at 72 h, up-regulation of pathways related to “transcription regulatory region nucleic acid binding”, “regulatory RNA binding”, “response to other organism”, “regulation of DNA repair” and “inflammatory response” were observed. At 120 h, molecular function pathways were up-regulated, including “Toll-like receptor binding” and “5′-3′RNA helicase/exonuclease activity”, while among BP, “Biological process involved in interspecies interaction between organisms” was also enriched. Still, in the gills, the few negatively regulated genes (Figure 4B, Supplementary File S1) contributed to the enrichment of some MFs at 72 h post-infection, including “signalling receptor regulator/activator activity”, “immune receptor activity”, “cytokine receptor binding/activity” and “chemokine receptor binding/activity”.

In the HK, at 72 h, the number of positively immune-related enriched pathways was significantly superior compared to the remaining tissues (Figure 4C, Supplementary File S1). Molecular functions such as the “Hsp70/heat shock protein binding”, “cytokine receptor binding”, and “tRNA metabolic process/aminoacylation for protein translation/aminoacylation” and BPs such as “response to heat” and “amino acid activation” were found to be enriched. At the last sampling point (120 h), a smaller number of positively regulated pathways were observed, with exclusive enrichment of the molecular function for “lysozyme activity”, “chemokine receptor binding/activity”, and the BP “defense response to virus” and “biological process involved in interspecies interaction between organisms”. In the present tissue (Figure 4D, Supplementary File S1), only the enrichment of negatively regulated pathways was observed at 120 h, with strong regulation of the molecular functions: “sequence-specific DNA binding”, “RNA polymerase II transcription regulatory region sequence-specific DNA binding” and “peroxidase activity”. In addition, the down-regulation of the biological processes “transcription by RNA polymerase II”, “homeostasis of number of cells” and “erythrocyte homeostasis/differentiation” was observed.

The spleen was the tissue with the highest number of enriched immune-related pathways, which also contained the largest number of DEGs among the tissues analyzed Figure 4E, Supplementary File S1), and the only tissue where enriched pathways were observed at 24 h after infection, displaying a response related to “chemokine receptor binding” and “cytokine/chemokine activity”. Moreover, it can be perceived from the bubble chart that there was a stronger response at 72 h compared to other times, particularly in important molecular function pathways such as “RNA binding”, “Hsp90/70 protein binding”, “cytokine/chemokine receptor binding”, and “cytokine/chemokine activity” and BPs such as “leukocyte mediated immunity”, “immune system process/response” “immune effector process” and “gene expression”. The latter pathway has the highest number of DEGs. Processes related to the response to a viral infection such as “response to virus” and “defense response to virus” were also found to be up-regulated at 72 h and maintained at 120 h. Additionally, at a later sampling point (120 h), an enrichment of pathways related to RNA processes such as “regulatory RNA binding”, “miRNA binding” and “double-stranded RNA adenosine deaminase activity”, as well as “phagocytosis”, was observed. Finally, the enrichment analysis of the negatively regulated DEGs in the spleen (Figure 4F, Supplementary File S1) showed a down-regulation of “immune system process” and “immune response” at 72 h followed by a down-regulation at 120 h of “cell surface receptor signalling pathway”.

#### 3.4.3. Common and Unique DEGs

**Figure 5:**
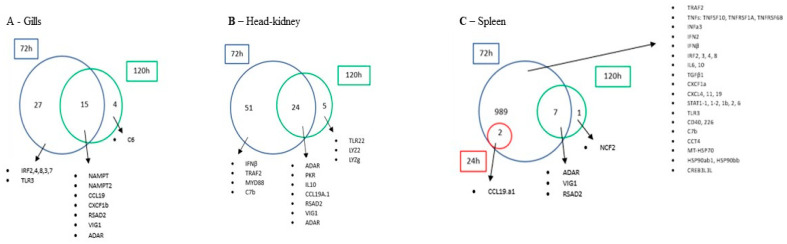
Venn diagrams showing the number of common and unique genes up-regulated in gills (**A**), HK (**B**) and spleen (**C**), up-regulated at 24, 72 and 120 h post-infection.

**Figure 6:**
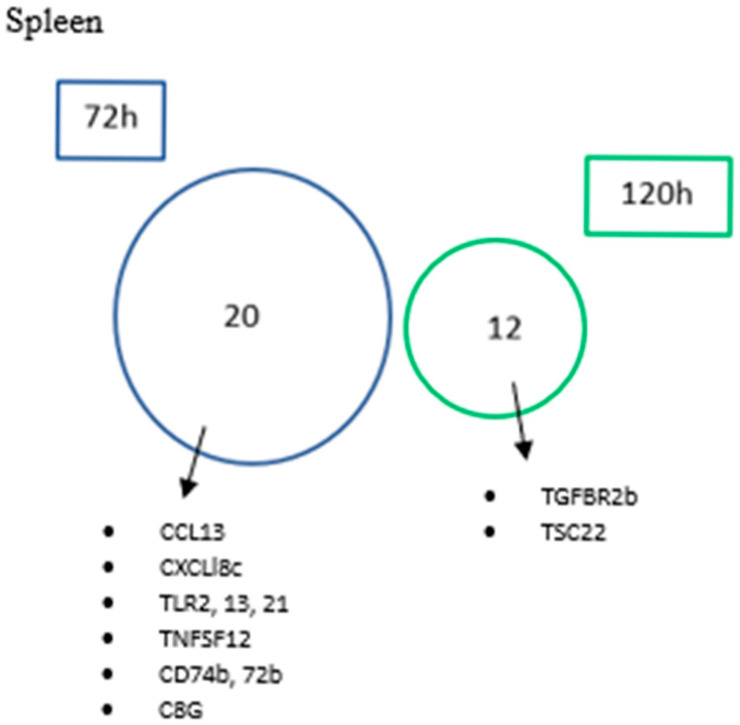
Venn diagram showing the number of common and unique genes down-regulated in spleen at 72 and 120 h post-infection.

In order to better interpret the data, a comparative analysis was performed for each tissue to assess the number of commonly and uniquely expressed up-regulated (Figure 5) and down-regulated genes (Figure 6) across different sampling times. As anticipated, the low number of DEGs observed at 24 h across all tissues resulted in no commonly expressed genes being identified at this time point when compared to the other time points. The lack of DEGs in gills and HK (120 and 72 h, respectively) also did not allow the identification of genes.

In the gills (Figure 5A), a total of 15 genes were commonly up-regulated at both 72 and 120 h, including NAMPT, NAMPT2, CCL19, CXCF1b, RSAD2, VIG1 and ADAR. In regard to exclusively up-regulated genes in this tissue, at 72 h, a total of 27 genes were found (i.e., IRF2, 3, 4, 7 and 8 and TLR2) while at 120 h, only 4 exclusively up-regulated genes were found, such as the C6.

In the HK (Figure 5B), a total of 24 genes were commonly expressed at 72 and 120 h, highlighting ADAR, PKR, IL10, CCL19A.1, RSAD2, VIG1 and ADAR. At the peak of infection (72 h) fifty-one genes, such as IFNβ, TRAF2, MYD88 and C7b, were exclusively up-regulated. At the last sampling point (120 h), only five DEGs were found to be exclusively expressed (e.g., TLR22, LYZ2 and LYZg).

The spleen (Figure 5C) was shown to be the tissue with the highest number of exclusively up-regulated genes at 72 h, with a total of 989 exclusive genes, with an emphasis on TRAF2; TNFs TNFSF10, TNFRSF1A and TNFRSF6B; IFNa3; IFN2; IFNβ; IRF2, 3, 4 and 8; IL6 and 10; TGFβ1; CXCF1a; CXCL4, 11 and 19; STAT1-1, 1-2, 1b, 2 and 6; TLR3; CD40 and 226; C7b; CCT4; MT-HSP70; HSP90ab1; HSP90bb; and CREB3L3L, while two of these genes were also up-regulated at 24 h. Moreover, at 120 h, only one exclusive DEG was observed, the NCF2. More importantly, a total of seven genes were found commonly expressed at 72 and 120 h (e.g., VIG1, RSAD2 and ADAR). Interestingly, down-regulated genes were only observed in the spleen (Figure 6). However, no common genes were identified at different times after infection. At 72 h, a total of 20 genes were exclusively down-regulated, highlighting CCL13, CXCLl8c, TLR2, 13, 21, TNFSF12, CD74b, 72b and C8G, while at 120 h, twelve genes were found down-regulated (e.g., TGFBR2b and TSC22).

## 4. Discussion

In this study, the immune response of rainbow trout to VHSV infection was characterized across multiple tissues, with the aim of uncovering the host mechanisms involved in disease development, both systemically and locally, through a holistic approach.

### 4.1. Route of VHSV Entry

At the outset of the discussion, it is crucial to define the peak of infection observed in this study, which is marked by the highest pathogen load in the host tissues at 72 h post-infection in trout infected via bath immersion. This is clearly displayed in the gills, liver, HK, spleen and skin, where the amount of viral particles reached the maximum level, remaining high after this time. A study by Garver et al. [43] in Atlantic salmon (*Salmo salar*) identified 5 × 10^6^ copies µL^−1^ as a high viral load of VHSV in the HK. Despite this finding, no further evaluations of VHSV viral load in fish tissues were conducted to date. Although no significant differences were observed between sampling points, the skin of infected trout presented a consistently high number of viral copies, with 10^6.69^ copies µL^−1^ early in the infection (24 h), slightly increasing in number up to 10^6.97^ copies µL^−1^ at 72 h. The quick and strong rise in the viral copies in this tissue may suggest that skin is the main target of the virus and the preferable route of entry in the host. In addition to the skin, the gills, as an external organ directly in contact with the fish environment, may play an important role in the dissemination of VHSV, allowing its absorption and replication [14,15,44]. In fact, Wolf [45] described both gills and skin as the first entry route for the virus, which then uses the bloodstream to reach other tissues [15]. This is confirmed in the present study, with viral loads detected in the skin and gills as early as 24 h post-infection (10^4.34^). A peak in infection was then observed at 72 h, with viral copy numbers reaching 10^6.97^ in the skin and 10^7.12^ in the gills. By 120 h, viral loads slightly decreased to 10^5.88^ in the skin and 10^6.85^ in the gills.

VHSV active replication can cause severe hemorrhages and reach important organs of the immune system, such as the HK, liver and spleen, with the spleen being clearly defined as a target of the pathogen [17,45,46]. Although no statistically significant differences in viral load were observed in the HK, spleen or gills between 72 and 120 h post-infection, the data suggest ongoing immune activation, as indicated by the sustained transcription of pro-inflammatory cytokines such as IL-6 and anti-inflammatory IL-10, along with several chemokines during this period, which is therefore expected according to Cuesta and Tafalla [22]. In opposition, gut tissue did not show a clear modulation of the viral cDNA copy number throughout the time of infection, while presenting a constantly low viral load, suggesting that this pathogen does not replicate as actively in this organ.

### 4.2. Cell Antiviral Receptors

In viral infections, the detection of the pathogen in the cytoplasm of host cells triggers a signaling cascade involving multiple intracellular molecules, ultimately leading to the activation of an antiviral immune response [47,48]. In particular, immune-competent cells possess specialized pattern recognition receptors (PRRs) that are able to detect viral-associated molecular patterns [49,50,51]. More specifically, members of the Toll-like receptor (TLR) family and cytosolic retinoic acid inducible gene I (RIG-I)-like receptor (RLRs) family, such as RIG-1, MDAS and LGP2 [52,53,54,55], act as sensors for the recognition and signalling of viruses in the cell cytoplasm [56]. When the viral ssRNA is detected, TLRs, in conjunction with the MyD88 protein—an essential and universal adapter in viral infections that mediates signal transduction for all TLRs [57]—along with RIG-I-like receptors (RLRs) in the cytoplasm, trigger a signaling cascade. This cascade activates the transcription of several genes, ultimately leading to the activation of type I IFNs, which are key components of the innate immune response [51,58]. In the present study, at the peak of infection (72 h), specific viral receptors, such as a receptor for dsRNA, encoded by the TLR3 gene, generated during most viral infections [59], was found to be positively expressed in the gills and spleen, enriching pathways such as the “immune response”, “biological process involved in interspecies interaction between organisms” and “response to another organism”. Previous studies demonstrate that *tlr3* is involved in the activation and recruitment of antiviral genes to the site of infection [60]. In the HK at 120 h, *tlr22* was up-regulated, and enriched in the same pathways as *tlr3* and additionally in pathways related to the biological process, “defense response” and “innate immune response”. This gene, like the previous one, is a fish-specific TLR, which acts as a crucial detector in a viral infection and dsRNA, initiating signaling and innate immune response [61].

Only a negative expression of *tlr2*, *13* and *21* was observed at 72 h in the spleen associated with “biological mechanisms of immune response”. The *myd88*, an adapter in the transduction of TLRs, was found to be up-regulated only at 72 h in the HK, being associated with the enrichment of GO terms related to biological processes, namely “interaction between organisms” “immune/defense response” and “response to other organism”. This finding may suggest that the host only recognizes, at the systemic level, a marked presence of the virus at 72 h—characterized as the peak of infection. This is supported by the significant expression of genes involved in detecting and recognizing the presence of the virus [56], as observed by the expression of *tlr3* in the gills and spleen, which works together with the *myd88* adapter. In the present study, the viral load in the HK was still significantly elevated at 72 h and remained elevated at 120 h, and the positive expression of *tlr22* at the last sampling point may suggest that there is still recognition/detection of the virus.

### 4.3. Interferon Inducible Genes—The Central Role of Viperin

Upon viral recognition, the association of the mitochondrial signaling protein (MAVS) with tumor necrosis factor (TNF) receptor-associated factor (TRAF), activates/facilitates the cytoplasmic kinase TANK-binding Kinase 1 (TBK1) [54,56], promoting the phosphorylation in the C-terminal serine-rich region of the IFN regulatory factor (IRF3/7) [51,62]. TRAF2 is known to be a mediator of several signaling pathways, such as cell death, inflammation and cell proliferation [63,64,65]. This gene is also involved in the innate immune response against pathogenic infections through type I IFN signaling [63,64,66,67,68]. An up-regulation of the expression of the *traf2* gene was observed at 72 h in HK and spleen within the “cytokine receptor binding” pathway, showing that at the peak of infection, these two important immunological organs are able to mount a faster and pro-inflammatory response to VHSV. Early-phase IFNs are mainly regulated by IRF3 and late-phase IFNs by IRF7 [62,69]. VHSV is highly sensitive to IFN I responses from host cells at an early stage of replication [70,71,72]. The IRF3 activation is considered the main regulator of IFN I in a viral infection [51,73], and this allows this protein to be transferred to the cell nucleus, binding to the IFN promoter and finally initiating its transcription [51,74]. Recent studies have investigated the role of the up-regulation of the *adar* gene, which can modulate various immune responses, particularly through the activation of interferon-associated pathways, such as that involving IRF3 [75]. In the present study, up-regulation of this gene was observed at 72 and 120 h in the gills, HK and spleen, suggesting that its activity may directly influence the course of infection and contribute to the host’s antiviral response against VHSV. Inside the cell nucleus, some ISGs, such as Myxovirus (Mx), Viperin (VIG/RSAD2) and ISG15, are proteins with direct antiviral function and important as responses against VHSV [20]. Together with IRF3, these proteins [20,31,62] promote virus degradation [51,76,77,78]. In this way, an antiviral state is produced within the cell nucleus, which releases an innate immune response against VHSV. In this study, a positive expression of several IRFs at 72 h, namely *irf3* and *irf7* in the gills and spleen (both enriched in transcription regulatory region nucleic acid binding molecular function pathway) and *irf3* in the spleen, are genes enriched in the pathway involved in gene expression. Viperin, annotated as *rsda2* and *vig1*, was found to be up-regulated in all tissues, both at 72 h and 120 h, contributing to the enrichment of distinct pathways involved in immune response, such as “response/defense response to other organism”, “biological process involved in interspecies interaction between organisms”, “immune system process/immune response” and “defense/response to virus”. Additionally, at 72 and 120 h, this gene was up-regulated in pathways related to antiviral response—“defense response to virus” and “response to virus”—in the gills, HK and spleen. This points to the activation of *irf3/7* and *vig1* in order to decrease and degrade VHSV particles through their direct antiviral action at the peak of infection (72 h). Similarly, Angsujinda et al. [79] and Vaz et al. [48] observed a tendency for increased *irf3* in response to Betanodavirus infection, supporting its role in activating IFN I during infections with inhibiting viral replication. Moreover, several studies report that viperin can be associated with a positive amplification of the innate immune antiviral response, positively regulating IFNs [31]. The continued action of *viperin* (annotated as *vig1*) at 120 h in all tissues supports the importance of this gene in the sustenance of an antiviral state against VHSV, even after the peak of infection. In fact, some studies have highlighted the key role of viperin activity in antiviral response [31,80,81] while revealing its broad-spectrum antiviral activity across fish species and cell types [31]. For example, in humans, viperin allows the release of the Influenza A virus from the plasmatic membrane cells, due to its antiviral action against ssRNA virus [31,82]. In fish, viperin has similarly demonstrated antiviral capacity by inhibiting the replication of the SGIV virus (Singapore grouper iridovirus) in *Epinephelus coioides* [83], promoting resistance to megalocitivirus infection in *Oplegnathus fasciatus* [84], and significantly reducing VHSV transcription and replication in *Liza haematocheila* [31,85]. In rainbow trout, *viperin* is described as an inducible gene in leukocytes in response to VHSV [20], while its ability to reduce the replication of different viruses, such as spring viremia carp virus (SVCV) and Singapore grouper iridovirus (SGIV), was observed by ultimately facilitating the activation of an innate immune response via the IFN system through the RIG-I-like receptor (RLR) signaling pathway and improving the expression of ISGs and the overall inflammatory response [79,86]. While the first viperin homolog was discovered in rainbow trout [31], no further studies have been carried out on the matter in this species. Viperin antiviral activity is believed to stem from its interaction with viral RNA, specifically through the synthesis of the antiviral ribonucleotide 3′-deoxy-3′,4′-didehydrocytidine triphosphate (ddhCTP), which binds to RNA-dependent RNA polymerase (RdRp) and inhibits viral RNA synthesis [28,87,88,89,90]. Consequently, viperin is able to target ssRNA viruses, such as VHSV, and block both transcription and replication [89]. However, recent studies by Rivera-Serrano [91] have highlighted additional functions of viperin beyond its role as an antiviral ribonuclease. New evidence indicates that viperin also seems to be responsible for enhancing antiviral response and innate immune signalling by interacting with numerous cellular and viral proteins within the cell and acting in the degradation of ubiquitin-dependent viral proteins [28,89,90]. These multi-faceted mechanisms underscore the importance of viperin in antiviral defense, especially as recent findings in mammals emphasize its key role in innate immunity. Furthermore, viperin broad-spectrum antiviral activity has made it a subject of significant research across different species and cell types, including fish. Recent studies underscore the importance of viperin action against VHSV in fish using zebrafish (*Danio rerio*) as a model organism [89].

### 4.4. Cytokine Response

Additionally, viral infections also trigger the expression of inflammatory cytokines and chemokines, important factors in the resistance of various viral and bacterial infections, regulating inflammation and subsequent cell apoptosis [34,48,92]. Accordingly, in the present study, it was observed that at 72 h, the spleen presents positive expression of several chemokines (*cxcf1a*, *cxcl4*, *cxcl11*, *cxcl19*) enriched in pathways related to the inflammatory response, namely “cytokine/chemokine receptor binding”, “cytokine/chemokine activity”, “immune system process” and “immune response”, and also several pro-inflammatory cytokine (*il6* and *il10*), which, in addition to also being present in the pathways where chemokines were observed, were present in GO terms involved in biological processes, namely “gene expression” and “immune effector process”. Moreover, the presence of TNF genes was confirmed in the spleen (e.g., *tnfsf10*, *tnfrsf1a*, *tnfrsf6b*), leading to the enrichment of pathways related to immune response and cytokines, such as “cytokine receptor binding”, “immune system process” and “immune response”. A strong response in the spleen at the peak of infection was visible, suggesting an acute-phase pro-inflammatory response at the peak of infection, while no response was observed at 24 h. In the acute phase response, *il6/10* are induced by TNF, as confirmed by the joint expression of these genes. The elevated plasma NO from 24 h after the challenge until the last sampling point suggests that from the initial moments of the infection, it acted as a response to pro-inflammatory cytokines and TNFs, although genes were only observed to be regulated at the peak of infection in the spleen. The action of NO may have triggered cytokines, thus helping to control infection over time [34,48,93,94]. On the other hand, positive expression of chemokines (e.g., *cxcf1b*, *ccl19a.1*, *ccl19*, *cxcl11*) was also observed at 72 h and 120 h in gills and HK with enriched mechanisms and associated with “immune response”, “signaling receptor activator/regulator activity” and “cytokine/chemokine activity”, indicating that the pro-inflammatory activation of these molecules remains beyond the acute phase in order to help fight the infection. In the spleen at 72 h, there was a negative regulation of chemokines (e.g., *cxcl8c* and *ccl13*) and TNF (*tnfsf12*) both involved in “immune response” and “immune system process”, confirming the intensity of the infection in this tissue (target) and some exhaustion in the attempt for a pro-inflammatory response. In the study by Zhang [83], in grouper, *viperin* positively regulated the expression of cytokines involving interferon and inflammatory cytokines such as *irf3*, *tnfa* and *il-6*, as observed in the present study.

In an infected state, the ultimate goal is to have an inflammatory profile that favors an antiviral response and for the cell to be in an antiviral state. This requires the production of an innate immune response by the host through the activation and induction of a response by type I Interferon [20]. In the present study, only in the spleen at 72 h was a clear response from several IFNs (e.g., *ifna3*, *ifn2* and *ifnβ*) observed, resulting in the enrichment of several BP, such as “cytokine receptor binding”, “cytokine activity”, “response/defense response to virus” and “gene expression”. This indicates that this tissue is a target of VHSV and underscores its importance as an immunological organ in viral infections. On the other hand, the positive expression of *viperin*, an important antiviral gene in rainbow trout, in the target organ—spleen—and also in the gills and HK, suggests that this gene is widely distributed in different organs and in the presence of a viral infection, acting with the aim of minimizing the damage caused by the pathogen.

### 4.5. Trout Peripheral Antiviral Response

Finally, at the peripheral level, the response to VHSV infection is rapidly perceived by the decrease in RBCs and hemoglobin concentrations at 24 h, together with a decrease in hematocrit values at 120 h. This supports the impact of VHSV infection on the fish oxygen transport system of the fish bloodstream and a possible occurrence of anemia in response to the infection, as observed by Ferreira et al. [95]. In accordance, in the HK at 120 h, the “erythrocyte homeostasis/differentiation” pathways were down-regulated, confirming a possible occurrence of anemia caused by the viral infection. On the other hand, an increase in erythrocyte volume was observed at 120 h, which may be related to the increase and/or recovery of the fish’s metabolic activity [96], as the infection peak was at 72 h. Curiously, a general unresponsiveness of circulating leukocytes to VHSV infection is observed, with no modulation of the number of circulating neutrophils, while monocyte concentration remains irresponsive up to the 120 h sampling point. In contrast, thrombocyte numbers were found elevated until 72 h and then later decreased. During the development of an inflammatory process, a rapid response from the peripheral leukocytes is expected, as neutrophils are the first cells to migrate to sites of infection, followed by monocytes and thrombocytes [97,98]. In this study, no modulation of the peripheral neutrophils was observed, while monocytosis was observed only at 120 h, as observed in the study by Vaz et al. [48], with European sea bass (*Dicentrarchus labrax*) exposed to an infection with necrosis nervous virus (NNV). Moreover, the present results demonstrate thrombocytes’ high defense potential, as described by Stosik [97] with a significant high number at the peak of infection (72 h). Nonetheless, fish exposed to VHSV had a general lower number of lymphocytes compared to non-infected individuals. Such a lower response seems to be supported by other authors [48,99]. Regardless of time, the amount of plasma NO in infected fish remained high in relation to control animals, suggesting that the production of this compound in cells [100] throughout the infection may be involved in the inhibition of viral replication [101], as observed in the study by Vaz et al. [44]. NO may also have played a role as a vasodilator and increased cardiac output in compensation for the anemic condition, which could consequently deliver sufficient oxygen to the tissues. Nonetheless, supporting the observed attenuated peripheral leukocyte response, plasma lysozyme concentration was only found to be increased at 120 h, after the establishment of the virus at the systemic level. These results are supported by the significantly elevated expression of the *lyz2* and *lyzg* genes, which were enriched by the “lysozyme activity” pathway, as also observed in the HK (120 h). Moreover, while unresponsive throughout the initial sampling points, plasma peroxidase levels were shown to be significantly lower in VHSV-infected trouts compared to control individuals, which may point to an immunosuppression scenario [102,103,104]. In accordance, a down-regulation of the “peroxidase activity” pathway was found in the HK at 120 h, suggesting a possible inhibition of the host response, as the action of this enzyme allows the use of oxidative radicals to eliminate pathogens, acting as an important microbicidal agent [105].

## 5. Conclusions

Throughout the study, fish exhibited a general effort to combat infection. No host response or increase in viral load was detected at 24 h post-infection, indicating an incubation phase with limited early recognition.

The virus was confirmed to enter via external tissues (skin and gills), with peak infection at 72 h, evidenced by high viral load, altered plasma immune parameters, and strong up-regulation of antiviral genes, such as *viperin*. This gene maintained elevated expression until the end of the trial, confirming its critical role in the response to VHSV. A pronounced immune response in the spleen at 72 h also confirmed it as the virus’s main target organ. Although some immune activation was observed at 120 h, viral load remained high, suggesting that the resolution phase had not yet begun. Additional sampling beyond 120 h may clarify when recovery is initiated.

## Figures and Tables

**Table 1:** White blood cells (WBCs) (×10^4^ µL^−1^), red blood cells (RBCs) (×10^6^ µL^−1^), hematocrit (%), mean corpuscular volume (MCV) (µm^3^), hemoglobin (g dL^−1^), mean corpuscular hemoglobin (MCH) (pg cell^−1^), mean corpuscular hemoglobin concentration (MCHC) (g 100 mL^−1^), neutrophils, monocytes, lymphocytes and thrombocytes (×10^4^ µL^−1^) of rainbow trout challenged with VHSV and sampled 24, 72 and 120 h post-challenge.

Parameters	Treatment						Two-Way ANOVA							
	CTRL			VHSV			Time	Infection	Time × Infection	Time			Infection	
	24 h	72 h	120 h	24 h	72 h	120 h				24 h	72 h	120 h	CTRL	VHSV
WBCs	4.30 ± 0.71	3.60 ± 1.25	3.48 ± 1.02	3.44 ± 1.27	3.37 ± 0.83	2.87 ± 1.42	ns	ns	ns	-	-	-	-	-
RBCs	0.81 ± 0.11	0.67 ± 0.17	0.68 ± 0.17	0.68 ± 0.16	0.49 ± 0.05	0.5 ± 0.10	<0.01	<0.01	ns	^a^	^b^	^b^	^#^	*
Hematocrit	35.00 ± 0.00	37.33 ± 2.89	38.00 ± 1.58 ^#^	33.67 ± 3.21	26.00 ± 4.06	28.75 ± 3.50 *	ns	<0.01	0.041	-	-	-	-	-
MCV	450.93 ± 54.70	541.17 ± 62.70	539.92 ± 52.87	415.99 ± 123.75	531.83 ± 92.75	464.91 ± 15.19	0.05	ns	ns	^b^	^ab^	^a^	-	-
Hemoglobin	2.08 ± 0.26	1.57 ± 0.26	1.50 ± 0.20	1.50 ± 0.26	1.22 ± 0.23	0.81 ± 0.33	<0.01	<0.01	ns	^a^	^b^	^b^	^#^	*
MCH	25.29 ± 3.53	21.40 ± 3.16	19.96 ± 2.06	24.17 ± 7.97	24.83 ± 4.90	16.49 ± 3.21	ns	ns	ns	-	-	-	-	-
MCHC	5.78 ± 0.61	4.03 ± 0.27	3.81 ± 0.40	5.34 ± 0.98	4.36 ± 0.43	3.22 ± 0.39	ns	ns	ns	-	-	-	-	-
Neutrophils	0.15 ± 0.08	0.17 ± 0.03	0.12 ± 0.05	0.22 ± 0.17	0.09 ± 0.10	0.16 ± 0.13	ns	ns	ns	-	-	-	-	-
Monocytes	0.00 ± 0.00	0.00 ± 0.00	0.04 ± 0.03	0.00 ± 0.00	0.11 ± 0.05	0.19 ± 0.05	<0.01	<0.01	ns	^b^	^ab^	^a^	*	^#^
Lymphocytes	3.68 ± 0.69	2.98 ± 0.33	2.42 ± 0.77	2.40 ± 1.18	2.16 ± 0.93	2.26 ± 1.12	ns	0.03	ns	-	-	-	^#^	*
Thrombocytes	0.46 ± 0.33	0.99 ± 0.37	0.79 ± 0.50	0.70 ± 0.22 ^a^	0.72 ± 0.43 ^a^	0.21 ± 0.15 ^b^	ns	ns	0.03	-	-	-	-	-

Values represent means ± SD (n = 6). Different letters stand for significant differences attributed to time. Symbols stand for significant differences attributed to infection (CTRL vs. VHSV). * means the lowest value, and ^#^ means the highest values (multifactorial ANOVA; Tukey post hoc test; ns: non-significant; *p* ≤ 0.05). WBCs (white blood cells); RBCs (red blood cells); MCV (mean corpuscular volume); MCH (mean corpuscular hemoglobin); MCHC (mean corpuscular hemoglobin concentration); CTRL (control group); VHSV (infected group); ns (non-significant).

## Data Availability

The data used in this study are available in public and online repositories. Details regarding the repository/repositories and corresponding accession number(s) are provided at https://doi.org/10.6084/m9.figshare.29438105.v1 and https://doi.org/10.6084/m9.figshare.29438624.v1.

## Supplementary Materials

The following supporting information can be downloaded at https://www.mdpi.com/article/10.3390/biology14081003/s1: Figure S1: (A) Plasma antiproteases, (B) proteases and (C) bactericidal activity of rainbow trout challenged with VHSV and sampled 24, 72 and 120 h-infection (hpi). Values represent means ± SD (n = 6). Different letters stand for significant differences attributed to time. Symbols stand for significant differences attributed to infection. (Multifactorial ANOVA; Tukey post hoc test; ns: non-significant; *p* ≤ 0.05.) CTRL (control group) and VHSV (infected group). Table S1: *p*-values from two-way ANOVA for humoral immune response in plasma of rainbow trout challenged with VHSV and sampled 24, 72 and 120 h post-infection (ns: non-significant). Figure S2: Standard curve of the mean Ct value of each of the samples in real-time reverse transcriptase-polymerase chain reaction (RT-qPCR), according to the 10 in 10 dilutions of known VHSV TCID_50_ concentrations. Table S2: *p*-values from two-way ANOVA of viral quantification in skin, gills, gut, liver, head kidney (HK) and spleen of rainbow trout challenged with VHSV and sampled 24, 72 and 120 h post-challenge (ns: non-significant). Excel document file (Supplementary File S1): Supplementary file_RNAseq results for all data.
