# Supplementary material for: Unraveling the Systemic and Local Immune Response of Rainbow Trout (Oncorhynchus mykiss) to the Viral Hemorrhagic Septicemic Virus
Source: Biology (Basel). 2025 Aug 5;14(8):1003. doi: 10.3390/biology14081003 (PMC12383579; doi:10.3390/biology14081003)
Supplement: Supplementary file 1 [file biology-14-01003-s001.zip › biology-3760614-supplementary.pdf]

Supplementary material:

A

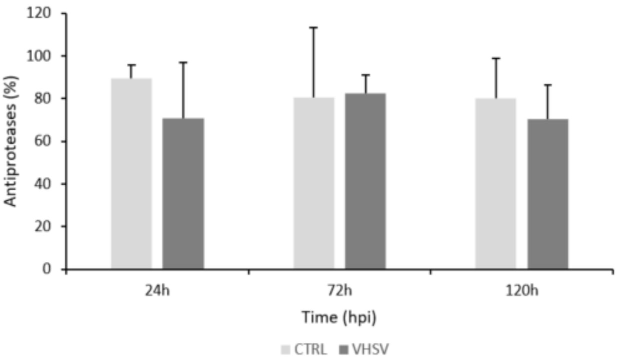

B

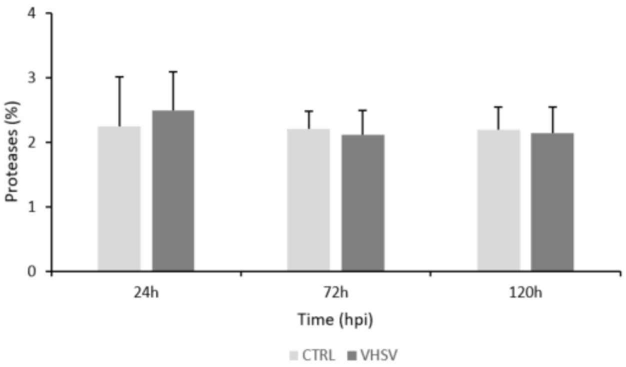

C

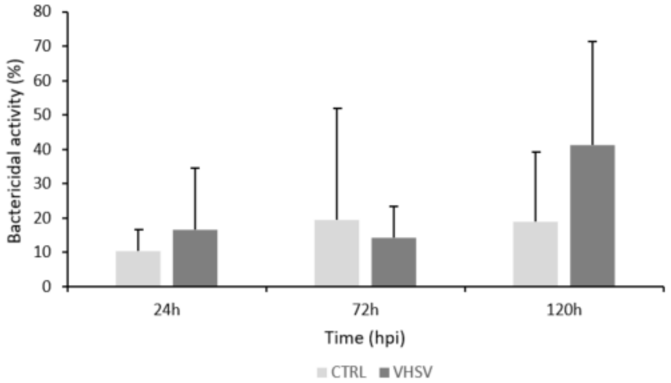

Figure S1: (A) Plasma antiproteases, (B) proteases and (C) bactericidal activity of rainbow trout challenged with VHSV and sampled 24, 72 and 120 hours-infection (hpi). Values represent means ± SD (n= 6). Different letters stand for significant differences attributed to time. Symbols stand for significant differences attributed to infection. (multifactorial ANOVA; Tukey post-hoc test; ns: non-significant; $p \leq 0.05$). CTRL (control group) and VHSV (infected group).

Table S1: *p*-values from two-way ANOVA for humoral immune response in plasma of rainbow trout challenged with VHSV and sampled 24, 72 and 120 h post-infection (ns: non-significant).

Parameters	Two-way ANOVA				
	Time	Infection	Time x Infection	Infection	
				CTRL	VHSV
Anti-proteases	ns	ns	ns	-	-
Proteases	ns	ns	ns	-	-
Bactericidal activity	ns	ns	ns	-	-
Lysozyme	ns	<0.01	<0.01	*	#

Nitric oxide	ns	<0.01	ns	*	#
Peroxidase activity	ns	ns	0.03	-	-

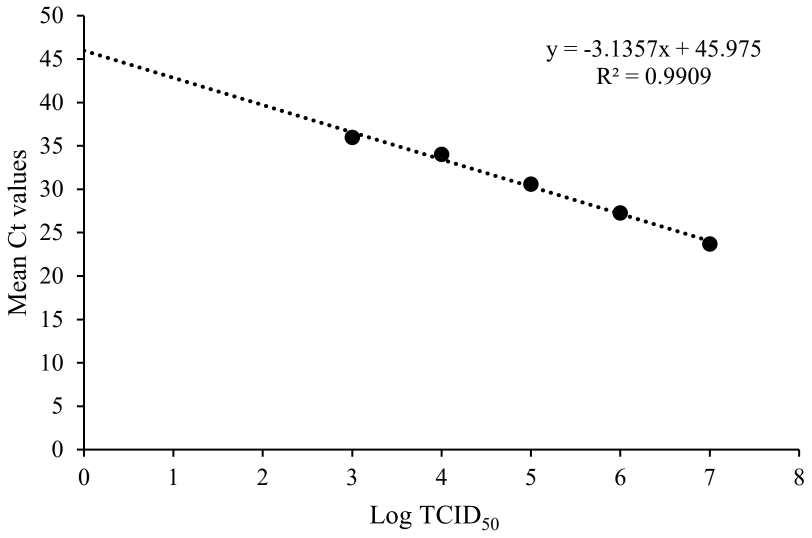

Figure S2. Standard curve of the mean Ct value of each of the samples in real-time reverse transcriptase-polymerase chain reaction (RT-qPCR), according to the 10 in 10 dilutions of known VHSV $TCID_{50}$ concentrations.

Y = -3.1357x + 45.975, with R^2=0.9909

Table S2: *p*-values from two-way ANOVA of viral quantification in skin, gills, gut, liver, head-kidney (HK) and spleen of rainbow trout challenged with VHSV and sampled 24, 72 and 120 h post-challenge. (ns: non-significant).

Parameters	Two-way ANOVA		
	Time	Infection	Time x Infection
Skin	ns	<0.001	ns
Gills	<0.001	<0.001	0.039
Gut	ns	<0.001	ns
Liver	<0.001	<0.001	<0.001
HK	ns	<0.001	0.021
Spleen	0.015	<0.001	0.028
